# Overnight Caloric Restriction Prior to Cardiac Arrest and Resuscitation Leads to Improved Survival and Neurological Outcome in a Rodent Model

**DOI:** 10.3389/fnins.2020.609670

**Published:** 2021-01-12

**Authors:** Matine Azadian, Guilian Tian, Afsheen Bazrafkan, Niki Maki, Masih Rafi, Nikole Chetty, Monica Desai, Ieeshiah Otarola, Francisco Aguirre, Shuhab M. Zaher, Ashar Khan, Yusuf Suri, Minwei Wang, Beth A. Lopour, Oswald Steward, Yama Akbari

**Affiliations:** ^1^Department of Neurology, School of Medicine, University of California, Irvine, Irvine, CA, United States; ^2^Department of Biomedical Engineering, University of California, Irvine, Irvine, CA, United States; ^3^Reeve-Irvine Research Center, School of Medicine, University of California, Irvine, Irvine, CA, United States; ^4^Department of Anatomy and Neurobiology, University of California, Irvine, Irvine, CA, United States; ^5^Department of Neurological Surgery, School of Medicine, University of California, Irvine, Irvine, CA, United States; ^6^Beckman Laser Institute and Medical Clinic, University of California, Irvine, Irvine, CA, United States

**Keywords:** cardiac arrest, caloric restriction, neurological recovery, cerebral ischemia, dietary restriction

## Abstract

While interest toward caloric restriction (CR) in various models of brain injury has increased in recent decades, studies have predominantly focused on the benefits of chronic or intermittent CR. The effects of ultra-short, including overnight, CR on acute ischemic brain injury are not well studied. Here, we show that overnight caloric restriction (75% over 14 h) prior to asphyxial cardiac arrest and resuscitation (CA) improves survival and neurological recovery as measured by, behavioral testing on neurological deficit scores, faster recovery of quantitative electroencephalography (EEG) burst suppression ratio, and complete prevention of neurodegeneration in multiple regions of the brain. We also show that overnight CR normalizes stress-induced hyperglycemia, while significantly decreasing insulin and glucagon production and increasing corticosterone and ketone body production. The benefits seen with ultra-short CR appear independent of Sirtuin 1 (SIRT-1) and brain-derived neurotrophic factor (BDNF) expression, which have been strongly linked to neuroprotective benefits seen in chronic CR. Mechanisms underlying neuroprotective effects remain to be defined, and may reveal targets for providing protection pre-CA or therapeutic interventions post-CA. These findings are also of high importance to basic sciences research as we demonstrate that minor, often-overlooked alterations to pre-experimental dietary procedures can significantly affect results, and by extension, research homogeneity and reproducibility, especially in acute ischemic brain injury models.

## Introduction

For almost a century, caloric restriction (CR) has been shown to have a multitude of health benefits in both humans and animals (Heilbronn and Ravussin, 2003). CR is defined as a reduction in caloric intake and can be daily, life-long, or intermittent. Many of the benefits appear to target aging, which includes prolongation of lifespan (Means et al., 1993; Mattson, 2003) and improvements in age-related deficits of learning (Hori et al., 1992) and memory (Hashimoto and Watanabe, 2005). In addition to its effects on aging, CR has been shown to be beneficial in various models of neurological diseases, most notably Alzheimer’s (Halagappa et al., 2007), Parkinson’s (Maswood et al., 2004), and epilepsy (Greene et al., 2002). Recently, CR has also been shown to have neuroprotective effects in various models of traumatic brain injury (Davis et al., 2008; Liu et al., 2017) and stroke (Manzanero et al., 2011).

Several studies on CR have proposed to explain its cellular and molecular mechanisms of action on the brain, which involve a wide range of pathways, including metabolic, inflammatory, oxidative stress, and cellular regenerative mechanisms (Ungvari et al., 2008; Maalouf et al., 2009). These studies, however, have predominantly focused on long-term CR, which is clinically impractical for a variety of reasons, including issues of adherence and of course, is irrelevant for treating brain injuries after they occur. On the other hand, brain injuries due to ischemic and some other insults trigger a cascade of degenerative processes that continue for hours and even days. As a result, more interest has been garnered toward short-term CR, which can range from days to months and has been shown to have both cardio- and neuroprotective benefits (Maalouf et al., 2009; Noyan et al., 2015). In one study, CR for 3 days prior to experimental stroke in a rodent model significantly reduced infarct volume (Varendi et al., 2014). Other studies have reported that CR for 14 days induced brain ischemic tolerance in a rodent model of middle cerebral artery occlusion via upregulation of SIRT-1 (Ran et al., 2015); and intermittent CR every 24 h for 3 months in rodents upregulated BDNF, and protected neurons against excitotoxic injury (Duan et al., 2001). Although long-term CR seems to involve a multiplicity of cellular pathways, it appears that short-term CR has thus far been linked to SIRT-1 and BDNF pathways, as several additional studies have pinpointed these downstream mechanisms, particularly in models of traumatic brain injury and focal stroke (Duan et al., 2001; Zuccato and Cattaneo, 2009; She et al., 2017).

We were interested in the effects of a simple overnight caloric restriction prior to global ischemic insult after an unexpected observation that this minor adjustment in our pre-experimental methods yielded noticeably improved recovery outcomes post-cardiac arrest. To our knowledge, there are no studies involving short-term caloric restriction of a single day or less. Pre-clinical analysis of such phenomena may provide simple and translatable approaches to potentially ameliorate recovery following focal ischemia, which affects over 795,000 people per year in the United States (Provencio et al., 2020), and global ischemia, which affects over 550,000 people annually in the United States (Hosseini et al., 2020). In this experiment, we induced global ischemia by cardiac arrest (CA) in rats that were calorically restricted (75%) overnight for 14 h and assessed for changes in outcome. In attempt to better understand such changes, we measured levels of glucose, insulin, glucagon, corticosterone, and ketone bodies in the blood, in addition to SIRT-1 and BDNF expression in the brain.

## Materials and Methods

### Animal Preparation

Adult male Wistar rats (Charles River Laboratories, Wilmington, MA) weighing 300–370 g were used in this study. No significant difference in weight was observed at any timepoint of the experiment. The animals were housed under standard conditions (23 ± 2°C, 60–70% relative humidity, 12 h light and dark cycles; free access to food and water). Animals typically arrived 2 weeks prior to experiments and were handled daily for 5 min to promote habituation and reduction of stress levels. To enable monitoring of electrocorticography (ECoG), 1 week prior to the experiment, each rat had two electrodes (1.57 mm in diameter) implanted on the dura (2 mm anterior and 2.5 mm lateral to bregma), corresponding to the left and right M1 motor cortices of the frontal lobes. Two additional electrodes were implanted (5.5 mm posterior and 4 mm lateral to bregma), corresponding to the left and right V1 visual cortices. A reference electrode was also placed 3 mm posterior to lambda over the cerebellum. All animal procedures were approved by the University of California Animal Care Committee (Irvine, CA) and conformed to the recommendations of the American Veterinary Medical Association Panel on Euthanasia.

### Dietary Restriction

One week after electrode implantation, rats were divided in two groups, control (*n* = 14) and calorically restricted (CR; *n* = 14). The control group had unlimited access to food during the entire experiment. Each rat from the CR group was fed 25% of the average daily food intake of the control group. Average daily food intake was calculated as weight of standard laboratory chow pellets consumed per day per rat. Each CR rat was calorically restricted overnight, starting at 6:00 p.m., approximately 14 h prior to surgical procedures at 8:00 a.m. on the following morning and 18 h prior to cardiac arrest at 12:00 p.m. Capillary blood ketone (β-hydroxybutyrate) levels were measured the morning after caloric restriction prior to surgical procedures with a Precision Xtra^®^ System (Abbott, Princeton, NJ). Rats in the CR group were allowed to resume *ad libitum* feeding during the recovery period following surgical and cardiac arrest procedures. Both groups had *ad libitum* access to water throughout the experiment.

### Cardiac Arrest Experiment

**FIGURE 1:**
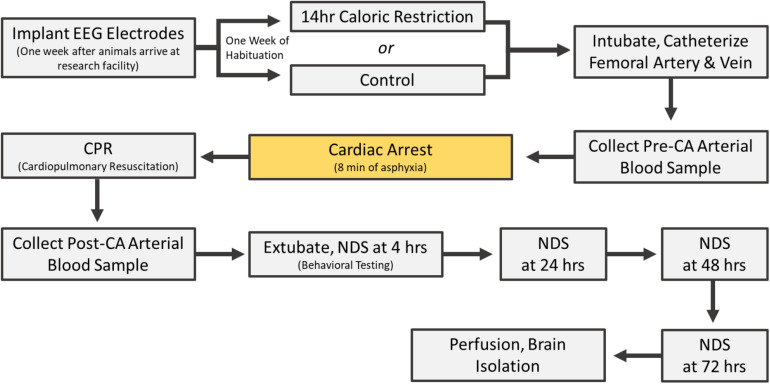
Cardiac arrest experimental timeline.

Rats underwent cardiac arrest one at a time (one experiment per day). All control experiments were completed in the same period; followed by a period of CR experiments. On the day of CA, rats were endotracheally intubated, connected to a TOPO^TM^ mechanical ventilator (Kent Scientific, Torrington, CT), and maintained under 2% isoflurane anesthesia carried by 50% O_2_ and 50% N_2_ gas during the surgical preparations leading up to CA. The femoral artery and vein were cannulated to monitor blood pressure and heart rate and to allow for the intravenous (i.v.) administration of medications. While under mechanical ventilation, positive end expiratory pressure was maintained at 3 cm H_2_O and body temperature was monitored with a rectal probe and maintained at 37°C. Cardiac arrest was induced via an 8 min duration of controlled asphyxia followed by cardiopulmonary resuscitation (CPR) until return of spontaneous circulation as previously described (Kang et al., 2017; Yin et al., 2019; Crouzet et al., 2020). No isoflurane anesthesia was administered during or after CPR for the remainder of the experiment. Approximately 250 μL of arterial blood was collected 10 min before asphyxia and 10 min after return of spontaneous circulation. Blood gas levels, in addition to blood glucose, was measured at both timepoints with an i-STAT^®^ System (Abbott, Princeton, NJ). Vessels were decannulated, and when spontaneous respirations were adequate, rats were extubated (see Figure 1 for experimental timeline). Physiological parameters including blood pH, calcium, potassium, pO_2_ and pCO_2_ were monitored during surgery. There was no significant difference in these parameters between CR and control groups either before or after CA (Supplementary Table 1).

### Post-cardiac Arrest Care

Normal saline (5 mL) and Ringer’s lactate (5 mL) was administered subcutaneously (s.c.) 5 h after CPR to limit dehydration until the rats resumed water consumption independently. Prophylactic cefazolin (45 mg/kg) was administered to limit any risk of infection. One-fourth cup of HydroGel (ClearH_2_O, Portland, ME) and 10 pellets of standard laboratory chow was soaked in water and placed near the rats’ mouth and throughout the cage until they recovered and resumed independent consumption of standard chow. At 24 h post-CA, capillary blood ketone levels were again measured. Rats were re-examined every 24 h thereafter to ensure proper hydration and food consumption. Both CR and the control group underwent analogous surgical, cardiac arrest, and post-cardiac arrest procedures. All rats were permitted *ad libitum* access to food and water following cardiac arrest.

### Neurological Evaluation

Experimental cardiac arrest causes neurological impairments during the early post-CA period with possible mortality. Accordingly, death following CR was one outcome measure (see section “Results”). Neurological recovery in surviving rats was quantified using the Neurological Deficit Scale (NDS; Table 1). The NDS consists of components that measure arousal, brainstem function, motor and sensory activities, as described previously (Kang et al., 2017; Wilson et al., 2019). Neurological recovery was evaluated at 4, 24, 48, and 72 h post-CA by well-trained personnel who were blind to dietary groupings.

**TABLE 1:** Neurological deficit scale scoring chart.

**Assessment**	**Component**	**Subscore**	**Total**
(A) Arousal	Consciousness	Normal *(10)* | Lethargic *(5)* | Comatose *(0)*	–/19
	Eyes	Open independently *(3)* | Open to Pain *(1)* | Absent *(0)*	
	Respiration	Normal (6,1 | Abnormal *(3)* | Absent *(0)*	
(B) Brainstem	Olfaction	Present *(3)* | Absent *(0)*	–/21
	Vision reflex	Present *(3)* | Absent *(0)*	
	Pupillary reflex	Present *(3)* | Absent *(0)*	
	Corneal reflex	Present *(3)* | Absent *(0)*	
	Startle reflex	Present *(3)* | Absent *(0)*	
	Whisker stimulation	Present *(3)* | Absent *(0)*	
	Swallowing	Present *(3)* | Absent *(0)*	
(C) Motor	Limbs*	Normal(3) | Weak(1) | No movement *(0)*	–/6
(D) Sensory	Pain response*	Brisk *(3)* | Weak *(1)* | No movement *(0)*	–/6
(E) Motor behavioral	Gait	Normal *(3)* | Abnormal *(1)* | Absent *(0)*	–6
	Balance on beam	Normal *(3)* | Abnormal *(1)* | Absent *(0)*	
(F) Behavioral	Righting reflex	Normal *(3)* | Abnormal *(1)* | Absent *(0)*	–/12
	Negative geotaxis	Normal *(3)* | Abnormal *(1)* | Absent *(0)*	
	Spatial awareness	Normal *(3)* | Abnormal *(1)* | Absent *(0)*	
	Turning alley	Normal *(3)* | Abnormal *(0)* | Absent *(0)*	
	*Tested and scored separately, arms only	Outcome: Best = 70 Worst = 0 **Total score = **	–/70

### ECoG Acquisition and Analysis

Electrocorticogram (ECoG) data was recorded during isoflurane administration, throughout the CA experiment, and continuously for 4 h post-CA. All signal processing was executed using MATLAB (The MathWorks, Inc., Natick, MA). A common average reference was used to reduce pervasive noise. The data was bandpass filtered from 1 to 50 Hz to remove any DC offsets and electrical noise. The signal was downsampled from 1,526 to 254 Hz to reduce computational load. Three rats in the CR group were excluded from ECoG analysis due to extensive EKG contamination. Burst Suppression Ratio (BSR) was calculated from 60 s windows with 30 s overlap (50%). Suppression was defined as intervals greater than 0.5 s during which the ECoG did not exceed ± 25 mV1. The BSR was then calculated as the fraction of the 60 s epoch that met the criteria for suppression (Rampil, 1998; Rampil et al., 1988). The mean BSR was taken across channels to obtain one value per epoch.

### Brain Tissue Collection

At 72 h following CA, surviving rats were anesthetized with sodium pentobarbital and perfused transcardially with 0.9% NaCl solution followed by 0.1 M phosphate buffered saline, pH 7.4. Brains were separated at the mid-sagittal plane into left and right hemispheres. The left hemisphere was post-fixed in 4% PFA for 24 h at 4°C and cryoprotected in 30% sucrose for 4 days. It was then frozen in optimal cutting temperature (OCT) embedding medium and stored in −80°C until sectioned. The right hemisphere was flash frozen in dry ice and stored in −80°C until homogenization for western blot analysis.

### Histologic Analysis

Left brain hemispheres frozen in OCT were coronally sectioned at 30 μm using a cryostat (Microtome HM 505N). Sections were stored in serial order in a 96-well plate in 1 × PBS with sodium azide at 4°C. Fluorojade-B (FJ-B) staining was used to scan and identify neuronal degeneration at 72 h post-CA. To conform to the stereological standards of systematic random sampling, sections from figure 10 (bregma, 3.24 mm) to figure 155 (bregma, −14.64 mm) in the rat brain atlas (*The Rat Brain in Stereotaxic Coordinates, 6th edition*) were chosen at a 330 μm interval (one of every twelve sections) covering a total 17,880 μm scanned area of potential neurodegeneration. Selected sections were stained for 20 min in 0.0004% Fluorojade-B solution (EMD Millipore, Billerica, MA, United States) after 10 min incubation with 0.06% potassium permanganate. Sections were screened under the microscope and those with Fluorojade-B positive neurons were marked for cell counting. To further avoid biased sampling, two additional sections were chosen at −90 μm anterior and +90 μm posterior of the original marked section.

### Cell Counting

Images of sections were obtained on a Nikon Eclipse Ti-E microscope (Nikon Corporation, Tokyo, Japan) under standardized conditions, including exposure time, gain, and resolution. Square sampling fields were numerated and placed on images to encompass areas with Fluorojade-B positive neurons. A random integer generator was utilized to select half of the numerated sampling fields for cell counting. Unbiased counting frames, modified from West (West, 2012), were used to manually count cells, in which Fluorojade-B positive neurons that were partially or entirely within the top and right borders and did not intercept the bottom or left borders were considered to be in the counting frame and tallied. All images were blindly analyzed by three trained personnel using ImageJ Plugin “Cell Counter” (NIH, Bethesda, MD).

### Measurement of Blood Serum Analytes

Arterial blood samples collected during the CA experiment were processed in EDTA-coated tubes with 25 μL aprotinin. After centrifugation (1,000 × g, 15 min), serum samples were aliquoted and stored at −80°C until use for measurement of analytes.

Serum concentrations of corticosterone, glucagon, and insulin were simultaneously determined by using a magnetic bead assay (Milliplex MAP Rat Stress Hormone/Metabolic Panel, Millipore, Billerica, MA, United States). All procedures were performed according to manufacturer’s instructions, at room temperature and protected from light. Samples were analyzed in a Luminex MAGPIX system (Millipore Sigma, Burlington, MA). Analyte concentrations were calculated using Analyst software (Millipore Sigma, Burlington, MA) with a five-parameter logistic curve-fitting method.

### Western Blot Analysis

Brain segments in the right hemisphere (cerebellum for SIRT-1 analysis; hippocampus for BDNF analysis) were sonicated in PBS containing Pierce Protease Inhibitor (Cat #88,665, Fisher Scientific, Hampton, NH), assayed for total protein concentration, and then mixed with SDS sample buffer. The resulting samples were resolved by SDS-PAGE (8% polyacrylamide for SIRT-1 analysis; 16% polyacrylamide for BDNF analysis) and transferred onto PVDF membranes. The antibodies used were: ECL^TM^ anti-rabbit IgG (Cat #NA 934-1 ml, GE Healthcare, Chicago, IL; 1:5,000 dilution), mouse anti-beta tubulin (Cat# E7, Developmental Studies Hybridoma Bank of University of Iowa, Iowa City, IA), IRDye 800cw donkey anti-rabbit (Cat #32,212, Li-Cor Biosciences, Lincoln, NE; 1:10,000 dilution). For BDNF analysis the primary antibody used was rabbit anti-BDNF N-20 (Cat #SC-546, Santa Cruz Biotechnology, Dallas, TX; 1:1,000 dilution). For SIRT-1 analysis the primary antibody used was rabbit SIRT-1 (Cat # 07-131, Millipore Sigma, Burlington, MA; 1:500 dilution). The immunoreactive bands were detected using Amersham^TM^ ECL Select^TM^ detection reagent (Fisher Scientific, Hampton, NH) according to the manufacturer’s instructions. Bands were analyzed with ImageJ (NIH, Bethesda, MD).

### Statistical Analysis

Data analysis was performed using IBM SPSS Statistics Software (V21; IBM Corporation, Armonk, NY) and GraphPad Prism (V6.0; GraphPad Software Inc., La Jolla, CA). Signal processing was executed using MATLAB (The MathWorks, Inc., Natick, MA). The Kolmogorov–Smirnov test was applied to evaluate normalcy of distribution. Group values that were parametric were reported as mean ± SD and non-parametric variables were reported as median and interquartile range, unless otherwise noted. Specific statistical tests utilized are noted accordingly in the results and figure legends below. Appropriate *post hoc* tests were used as noted. ^∗^*p* < 0.05; ^∗∗^*p* < 0.01, were considered significant.

## Results

### CR Induces Normoglycemia and Inhibits Stress-Induced Hyperglycemia

**FIGURE 2:**
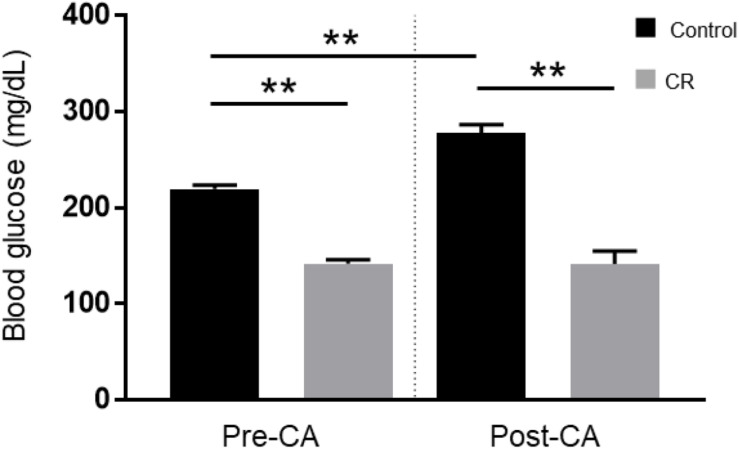
Arterial blood glucose of control vs. CR rats. Blood glucose was measured 10 min prior to CA and 10 min after resuscitation. While control (*ad lib*) rats demonstrate stress-induced hyperglycemia, CR significantly lowers and stabilizes blood glucose through the period of CA + CPR. **p < 0.01; by two-way repeated measures ANOVA. CA, cardiac arrest; CON, control; SEM, standard error of the mean.

To evaluate the effect of 14 h of CR on glycemia, a contributable outcome factor in countless models of brain injury, we measured arterial blood glucose levels 10 min prior to and after CA. As expected, surgical preparation for the CA experiment resulted in control (*ad lib*) rats exhibiting stress-induced hyperglycemia, with glucose levels above normal. However, as shown in Figure 2, the CR group exhibited lower blood glucose levels prior to CA (mean ± SD, 147.1 ± 15.7 mg/dL; *n* = 14) in comparison to control (219.5 ± 16.4 mg/dL; *n* = 14; *p* < 0.01). Assessment by two-way repeated measures ANOVA revealed significant differences between groups [*F*(1, 24) = 138, *p* < 0.01], and timepoints [*F*(1, 24) = 15.1, *p* < 0.01], and a significant interaction [*F*(1, 24) = 19.5, *p* < 0.01]. *Post hoc* comparisons by Sidak’s multiple comparisons test revealed that blood glucose levels remained stable after cardiac arrest in the CR group (143.4 ± 42.4 mg/dL; *n* = 14) but not in the control group (277.6 ± 30.6 mg/dL; *n* = 12), which exhibited a significant increase in glucose levels compared to pre-CA control values (*p* < 0.01). These results suggest that rats undergoing asphyxial CA display a stress-induced hyperglycemic response that is inhibited in entirety by 14 h of caloric restriction.

### CR Improves Survival After Cardiac Arrest

**FIGURE 3:**
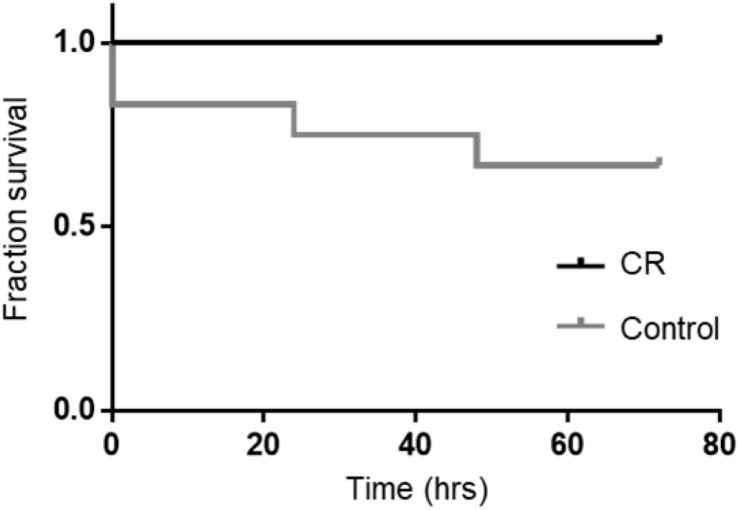
Fraction survival of post-CA rats. No mortalities occurred in the CR group. A log rank test (Mantel-Cox) was run to determine if there were differences in survival distribution. There was a statistically significant difference in survival distribution for the CR vs. control (*ad lib*) group; χ^2^(1) = 4.510, *p* < 0.05 by Kaplan-Meier survival analysis.

Based on our previous studies, we expected some mortality with an 8 min duration of asphyxia. In the control group, two rats could not be revived by CPR and an additional two died at 24 and 48 h post-CA. Remarkably however, all rats in the CR group were successfully resuscitated and survived the full term of experimentation (72 h). A Kaplan-Meier survival analysis (Kaplan and Meier, 1958) revealed that differences in survival were statistically significant [χ^2^(1) = 4.510, *p* < 0.05; Figure 3]. This suggests that 14 h of caloric restriction may have significant downstream cardioprotective and neuroprotective effects during global ischemic injury.

### CR Improves Neurological Recovery After Cardiac Arrest and Resuscitation

To evaluate neurological recovery post-CA of rats that were successfully resuscitated and survived, we measured NDS at 4, 24, 48, and 72 h post-CA. As shown in Table 1, NDS testing assesses arousal, brainstem reflexes, basic motor strength, withdrawal (sensation), gait, and primitive behaviors. In a pre-experimental, healthy state, rats have perfect NDS scores of 70, whereas post-CA all rats exhibit deficits in NDS.

**FIGURE 4:**
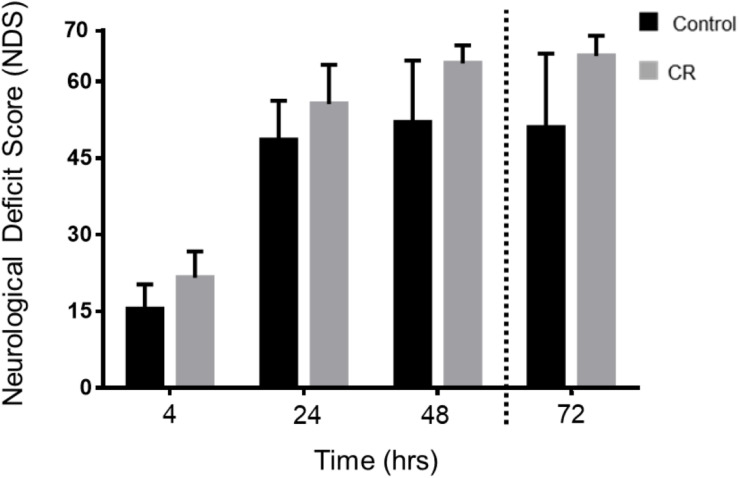
Neurological recovery of post-CA rats. NDS was measured at 4, 24, 48, and 72 h post-CA. Data is reported as mean ± SD at the first 3 timepoints given normal distribution of values. Two-way repeated measures ANOVA revealed an overall group difference and timepoint difference; and *post hoc* comparisons by Sidak’s multiple comparisons test revealed significant differences between groups at all 3 time points. 4 h [15.4 ± 4.9 (control *n* = 12) vs. 21.6 ± 5.1 (CR *n* = 14); *p* < 0.05], at 24 h [48.5 ± 7.7 (control *n* = 11) vs. 55.6 ± 8.0 (CR *n* = 14); *p* < 0.05], at 48 h [52.0 ± 12.2 (control *n* = 10) vs. 63.6 ± 3.6 (CR *n* = 14); *p* < 0.05]. Non-parametric Mann–Whitney *U*-test revealed differences in rank order NDS at 72 h. Data is reported as median, interquartile range. 72 h [51, 49.25–65 (control *n* = 10) vs. 65, 65–68 (CR *n* = 14); *p* < 0.05]. CA, cardiac arrest; CON, control; NDS, Neurological Deficit Scale; error bars for 4–48 h = SD, standard deviation; error bars for 72 h = interquartile range.

As shown in Figure 4, the CR group exhibited significantly higher NDS scores in comparison to the control group at every assessed timepoint post-CA. Two-way repeated measures ANOVA of the first 3 timepoints revealed a significant difference between groups [*F*(1, 49) = 19.22, *p* < 0.01] and timepoints [*F*(2, 49) = 148.2, *p* < 0.01]. Moreover, *post hoc* comparisons by Sidak’s multiple comparison test revealed significant differences between the control and CR group at the first 3 timepoints (*p* < 0.05). The 72 h timepoint had a left-skewed distribution of data, hence NDS scores were tested in rank by non-parametric Mann–Whitney *U*-test, and values were significantly different (*p* < 0.05, see figure legend for details). These results further suggest that a neuroprotective mechanism against ischemic insult appears to manifest as a downstream consequence of 14 h of caloric restriction, which in turn augments neurological recovery.

### CR Hastens Recovery of Quantitative ECoG as Measured by Burst Suppression Ratio After Cardiac Arrest and Resuscitation

**FIGURE 5:**
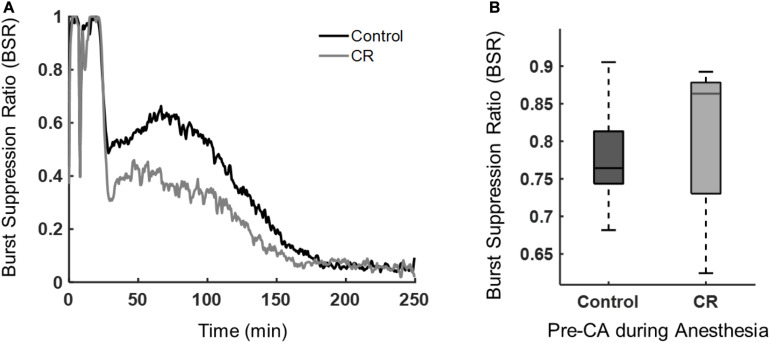
Burst suppression ratio of control vs. CR rats. **(A)** BSR beginning at asphyxia (time 0) until approximately 4 h post-CA. Repeated measures ANOVA revealed a significant group-time interaction, [*F*(432, 9,072) = 3.62, *p* < 0.01] when examining 30 min to the end of ECoG recording to exclude the dynamic period of asphyxia and CPR. **(B)** Median BSR over 5 min of pre-CA 2% isoflurane anesthesia period. No statistical difference was found between groups with a Mann-Whitney *U*-test (*p* = 0.60).

During anesthesia or after brain ischemia, burst suppression patterns (quasiperiodic high voltage periods followed with low or near zero voltages) on EEGs are commonplace (Ching et al., 2012). The BSR, a quantitative EEG feature, was used to characterize the amount of suppression present during anesthesia and during early recovery post-CA. Following asphyxia, the CR group had a lower mean BSR, indicating that these rats spent more time in bursting than suppression when compared to control rats. As shown in Figure 5, the BSR following CA had a significant time-group interaction as revealed by a repeated measures ANOVA [*F*(432, 9,072) = 3.62, *p* < 0.01]. While burst suppression is generally associated with poor prognosis after cardiac arrest, it has been reported previously that early increased bursting in rats after asphyxial cardiac arrest is linked to a good outcome (Geocadin et al., 2000). Also, a study in humans reported that a faster recovery of EEG from a bursting pattern to a continuous pattern after cardiac arrest can improve prognostication of a good neurological outcome (Cloostermans et al., 2012). Yet another study in humans showed patients with good outcomes had a significantly lower BSR during the first 48 h after cardiac arrest than patients with poor outcomes (Wennervirta et al., 2009). Our results revealed a similar pattern in the BSR when comparing the CR and control groups during the first 4 h after CA. Interestingly, during baseline recordings prior to cardiac arrest induction, while under 2% isoflurane anesthesia, there was no significant difference in anesthesia-induced BSR between groups, suggesting that both groups achieved a similar depth of anesthesia, regardless of the ultra-short 14 h caloric restriction, and hence a similar sensitivity of the brain to isoflurane in CR and non-CR animals.

### CR Reduces Neurodegeneration in Multiple Brain Regions

**FIGURE 6:**
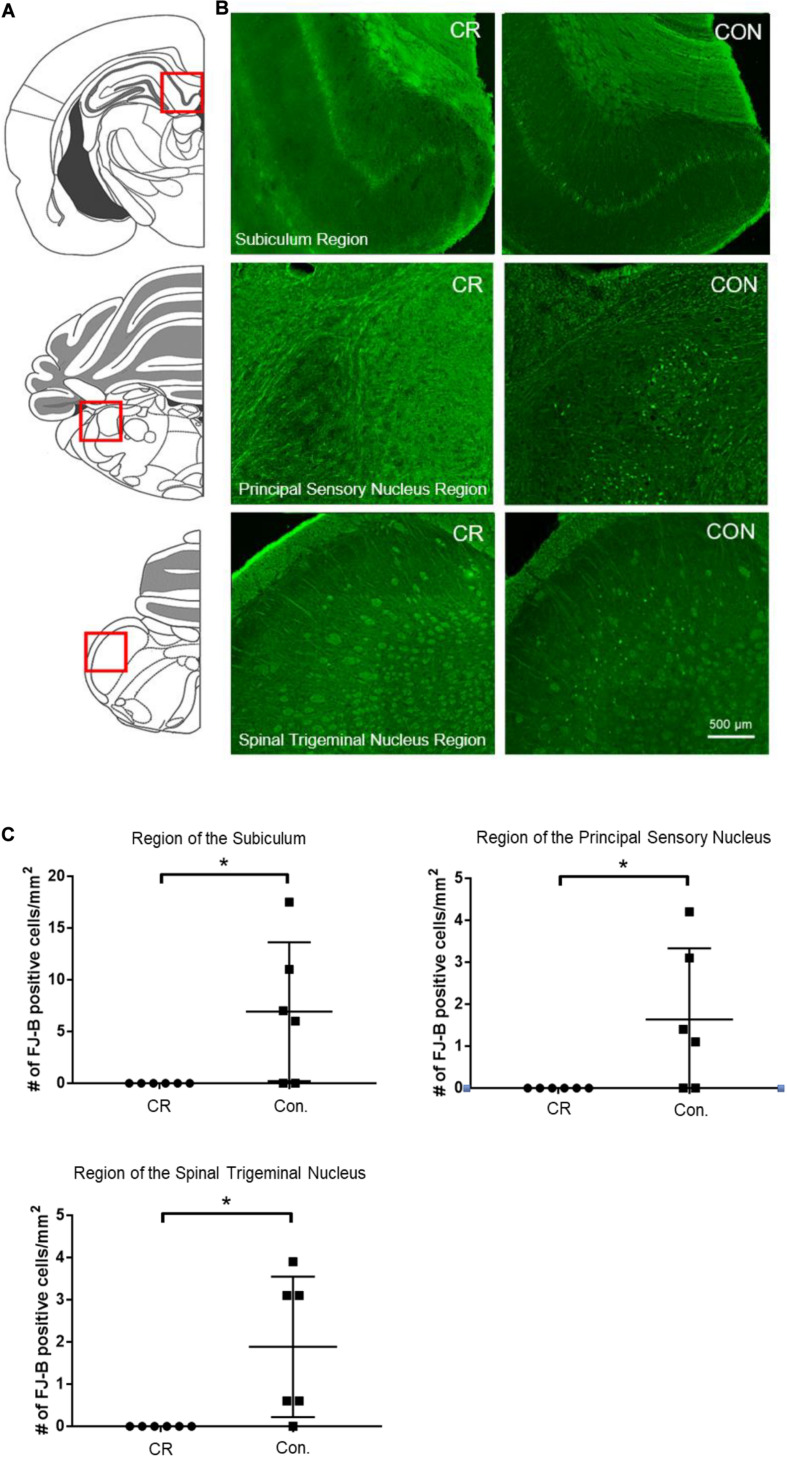
Neurodegeneration in multiple brain regions at 72 h post-CA. **(A)** Brain atlas. The region within the red square displays the physiological locales of neurodegeneration as shown in **(B)**. **(B)** Brain sections were stained with FJ-B; positive neurons are fluorescent (green). Images were cropped and signal intensities were adjusted linearly to be optimal for demonstration. **(C)** Number of FJ-B positive neurons were counted from 3 brain sections per region in each animal. The number of FJ-B positive neurons were significantly higher in control rats; CR rats exhibited no FJ-B positive neurons at these regions. Subiculum region *t* = 2.569; principal sensory nucleus region *t* = 2.376; spinal trigeminal nucleus region *t* = 2.875; *df* = 5, *p < 0.05 by unpaired *t*-test with Welch’s correction. CA, cardiac arrest; FJ-B, Fluorojade-B, CON, control; SEM, standard error of the mean.

To determine whether enhanced neurological function in CR rats was due to reduced neurodegeneration, we used Fluorojade-B (FJ-B) staining to identify degenerating neurons. Demonstrative of global cerebral ischemia, several regions throughout the brain were identified to have FJ-B positive neurons. Though both control and CR rats demonstrated FJ-B positivity in various brain regions, we found at least 3 regions that showed complete absence of neurodegeneration in the CR group: the subiculum, a region of the principal sensory nucleus in the thalamus, and a region of the spinal trigeminal nucleus. The subiculum, which is a component of hippocampal formation, has been linked to processes of spatial navigation and awareness (O’Mara et al., 2001); the principal sensory nucleus plays a role in sensation and proprioceptive feedback from whiskers and the muscles of the face (Schmid et al., 2016); and the spinal trigeminal nucleus receives sensory information from several cranial nerves (Haines, 2018). Images of the areas with FJ-B positive neurons were captured (as indicated by the red squares in Figure 6A and the representative images for each region in Figure 6B). FJ-B positive neurons were quantified in these regions (see section “Materials and Methods”). Remarkably, although FJ-B positive neurons were present in these areas in most control rats (Figure 6), there were zero FJ-B positive neurons in the same areas in the CR group (*n* = 6). Differences are statistically significant based on unpaired *t*-tests with Welch’s correction (see figure legend for details). These results suggest that 14 h of caloric restriction prior to ischemic insult leads to complete neuroprotective effects in defined areas of the brain that may play contributory roles in arousal, awareness, and functions that are vital to survival.

### CR Leads to Ketosis

**FIGURE 7:**
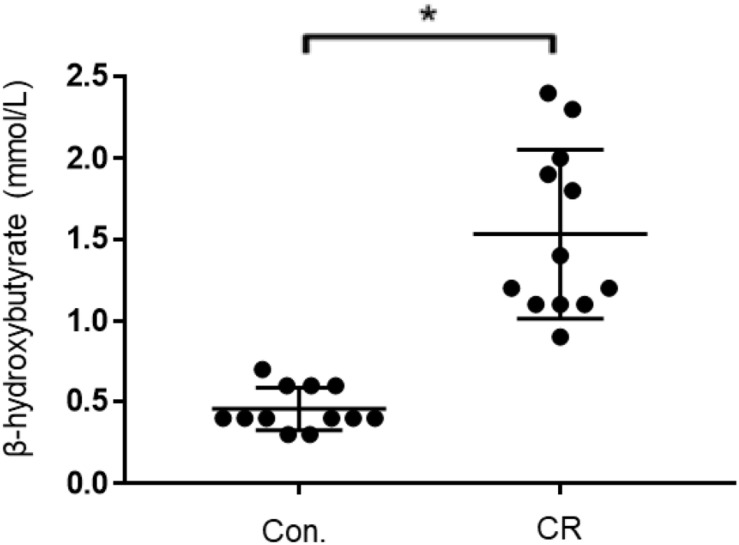
Capillary blood ketone (β-hydroxybutyrate) levels of control vs. CR rats. Blood ketones were measured after 14 h of caloric restriction. CR significantly increases ketone levels. *t* = 7.701, *df* = 26, ^∗^*p* < 0.01; by unpaired *t*-test with Welch’s correction. CR, caloric restriction; CON, control; SEM, standard error of the mean.

Given that both glycemia and caloric restriction are implicated in ketone body production, we measured capillary blood ketone levels (β-hydroxybutyrate) after 14 h of caloric restriction. As shown in Figure 7, the CR group exhibited significantly higher blood ketone levels (mean ± SD, 1.4 ± 0.5 mmol/L; *n* = 14) in comparison to control (0.6 ± 0.2 mmol/L; *n* = 14; *p* < 0.01) via unpaired *t*-test (*t* = 7.701, *df* = 26). These results indicate that 14 h of caloric restriction is of sufficient length to significantly increase the production of endogenous blood ketone levels.

### CR Leads to Higher Corticosterone and Lower Glucagon and Insulin

**FIGURE 8:**
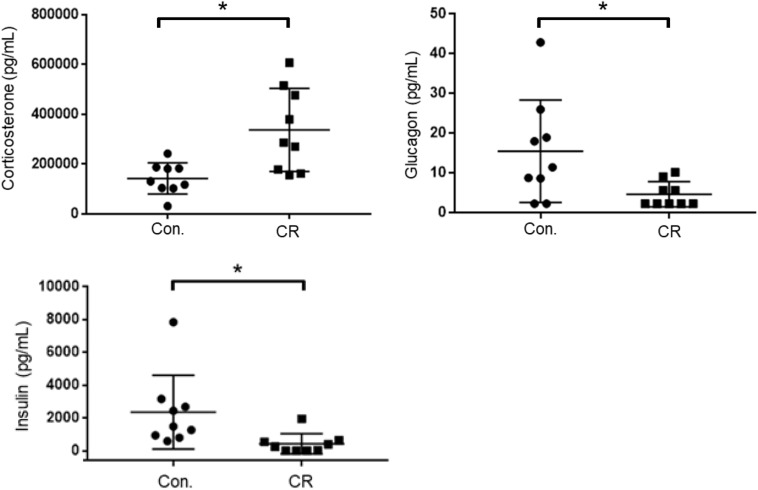
Arterial blood corticosterone, insulin, and glucagon levels of control vs. CR rats measured after 14 h of caloric restriction. CR significantly increases corticosterone levels and lowers glucagon and insulin levels. *p< 0.05; by unpaired *t*-test with Welch’s correction. CR, caloric restriction; CON, control; SEM, standard error of the mean.

As an indicator of stress, and to further elucidate upon the potential role of glycemia as a contributable outcome factor, we assessed corticosterone, glucagon, and insulin levels in arterial blood collected after 14 h of caloric restriction. As shown in Figure 8, the CR group exhibited significantly higher corticosterone levels in comparison to the control group [398,542 ± 59,024 pg/mL (*n* = 12) vs. 196,040 ± 22,092 pg/mL (*n* = 12); *p* < 0.05], likely indicative of a stress-response following metabolic deficits. Blood levels of glucagon, on the contrary, were significantly lower in the CR group in comparison to the control group [19.6 ± 7.8 pg/mL (*n* = 9) vs. 8.4 ± 2.9 pg/mL (*n* = 9); *p* < 0.05]. Blood levels of insulin were likewise significantly lower in the CR group in comparison to the control group [2,621 ± 965.4 pg/mL (*n* = 8) vs. 393.4 ± 265.9 pg/mL (*n* = 8; *p* < 0.05]. These differences are statistically significant based on unpaired *t*-tests with Welch’s correction (see figure legend for details).

### CR Does Not Change Expression of SIRT1 and BDNF

**FIGURE 9:**
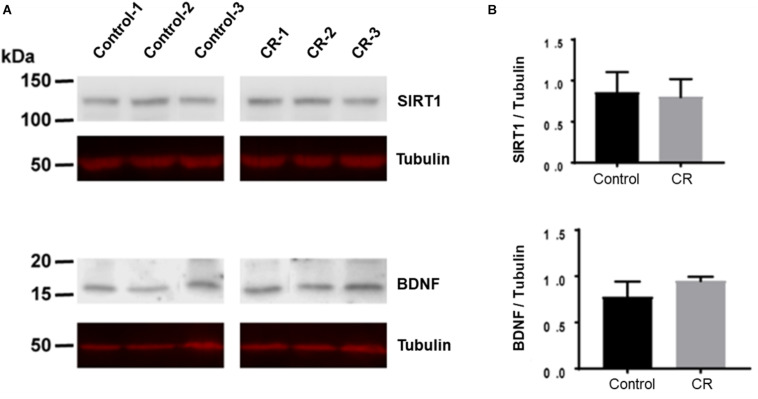
BDNF and SIRT-1 in brain homogenates following 14 h of CR. **(A)** The Western blot was probed first with SIRT1 antibody and showed no difference between CR (*n* = 3) and control groups (*n* = 3; *p* = 0.78) via unpaired *t*-test. It was then stripped and reprobed with an anti-tubulin antibody to confirm loading equivalence. **(B)** The Western blot was probed first with BDNF antibody and showed no significant difference between CR (*n* = 3) and controls (*n* = 3; *p* = 0.16) via unpaired *t*-test. It was then stripped and reprobed with an anti-tubulin antibody to confirm loading equivalence. Lane 1–3 are control samples, Lane 3–6 are CR samples.

Caloric restriction is known to upregulate brain-derived neurotrophic factor (BDNF) and sirtuin 1 (SIRT1) pathways in the brain, particularly following subacute periods of dietary restriction. To assess the potential upregulation due to 14 h of caloric restriction, we conducted Western blot analyses on brain homogenates of a separate cohort of rats that were calorically restricted for 14 h. Surprisingly, as shown in Figure 9, we found no significant difference in either BDNF or SIRT-1 expression via unpaired *t*-test after 14 h of caloric restriction in comparison to controls.

## Discussion

To date, many studies have indicated that CR paradigms exert robust protection against a multitude of diseases, including ischemic injury. Missing from the literature, however, is whether ultra-short (e.g., overnight) CR prior to a major ischemic insult provides protection. Such an ultra-short CR paradigm provides vastly more translational value, potentially introducing new therapeutic directions, while also having implications for experimental science and reproducibility. Our interests in CR arose from an unexpected observation in which a minor dietary adjustment prior to our experiment yielded noticeably improved recovery outcomes post-cardiac arrest. We therefore investigated the effect of a transient 14 h period of 75% overnight CR on outcome and neurological recovery after asphyxial CA, which leads to a non-shockable form of CA, now the most common type of cardiac arrest (Cobb et al., 2002; Herlitz et al., 2008; Goto et al., 2014; Keller and Halperin, 2015). Our results reveal improved survival, improved neurological recovery, and potent neuroprotection in specific regions of the rodent brain, including the subiculum, principal sensory nucleus, and spinal trigeminal nucleus. In attempt to dismantle the potential mechanisms involved at large, we explored various candidates that have been linked in prior studies with neuroprotection downstream of short-term caloric restriction. Our findings yield a normalization of blood glucose, increase in β-hydroxybutyrate and corticosterone, decrease in glucagon and insulin, and no significant upregulation of SIRT-1 and BDNF. Although further studies are warranted to pinpoint the specific mechanisms following overnight CR, to our knowledge, this is the first study showing significant benefit of overnight CR in an acute ischemic injury model. Below, we aim to briefly review the potential candidate mechanisms and related caveats in light of overnight CR potentially acting via an assembly of pathways that collectively convene to yield the noted effects of recovery in our model.

### CR-Induced Normoglycemia

Hyperglycemia has been widely shown to exacerbate various models of cerebral ischemia (Wass and Lanier, 1996; Kagansky et al., 2001; Niemann et al., 2011). In humans, hyperglycemia has been associated with significantly higher morbidity and mortality, and reduced long-term recovery following cardiac arrest (Wass and Lanier, 1996). Blood glucose levels have also been shown to significantly rise during CPR, possibly due to a stress-induced mechanism that leads to a deleterious hyperglycemic onset that further worsens neurological outcome (Longstreth and Inui, 1984).

In our model, rats exhibit slight stress-induced hyperglycemia after the surgical preparation needed to prepare for the CA experiment. Our findings reveal that a 14 h period of overnight CR is ample to blunt and in fact normalize this surgical stress-induced hyperglycemia prior to cardiac arrest induction. After CA + CPR, glucose levels rise to even higher levels in control rats, indicating exacerbation of stress-induced hyperglycemia, mirroring the stress-induced hyperglycemic phenomena noted in humans after CPR. Meanwhile, CR rats do not display any form of hyperglycemia either during the stress of surgical preparations or following CPR. It appears that a brief 14 h period of CR is sufficient to block the hyperglycemic response in our experimental paradigm. We cannot rule out whether the improvement in neurological outcome after CR is a result of normalization of blood glucose levels. However, it has been reported in both human and rodent studies that normalization of hyperglycemia by insulin administration did not improve neurological outcome after traumatic brain injury (Garg et al., 2006). We therefore postulate that the mechanisms underlying the neuroprotective effects of CR in our model go beyond those afforded by normoglycemia, warranting further experimental investigation.

### CR-Induced Ketosis

Global cerebral ischemia leads to a variety of deleterious effects, primarily due to a decrease in oxidative metabolism via a reduction of oxygen availability. Ketone bodies have been widely studied as neuroprotective molecules with promising potential to ameliorate downstream ischemic injury, such as those caused by lactate generation, apoptotic cascade activation, and free radical proliferation (Cobb et al., 2002). Administration of β-hydroxybutyrate in particular has been shown to prolong the survival time in rodent models of global cerebral ischemia, and to reduce infarct size following focal cerebral ischemia (Suzuki et al., 2002). Moreover, a ketogenic diet has been previously shown, through histological analysis, to provide neuroprotection in a rodent model of CA (Tai et al., 2008). In addition to neuroprotection in certain locales of the brain, 14 h of overnight CR induced a significant rise in endogenous β-hydroxybutyrate levels in our model, further supporting a possible causal relation between ketosis and neuroprotection in global cerebral ischemia. Of note, the blood-brain barrier (BBB) is relatively impermeable to ketone bodies unless they are transported by the carrier protein monocarboxylic acid transporters (MCT1 and MCT2). Although it is not well understood how MCTs are regulated, CR has been shown in various studies to increase the BBB uptake of ketone bodies by upregulating expression of MCT1 (Moreira et al., 2009). In addition, increases in MCT1 and MCT2 expression have been reported following ischemic cerebral injury (White and Venkatesh, 2011). Taken together, these studies support the hypothesis that the neurological recovery exhibited in our model of CA may, in part, be influenced by a β-hydroxybutyrate-mediated mechanism.

### Corticosterone, Glucagon, and Insulin

Cortisol is the primary glucocorticoid found in humans, whereas corticosterone is the dominant glucocorticoid in rodents. Glucocorticoids have been shown to have bimodal affects that may be beneficial during acute rises but harmful after prolonged elevation (Qiu et al., 2012). Additionally, they have both pro- and anti-inflammatory effects in various brain injury models (Sorrells and Sapolsky, 2007). They are believed to be potent inducers of apoptosis mainly through a shutdown of inflammatory response via inhibition of the NF-κB, a pro-inflammatory transcription factor (Roquilly et al., 2013). In other studies, they have been linked with deleterious effects that further exacerbate neurological recovery following brain injury (White-Gbadebo and Hamm, 1993). Given some of the deleterious effects of elevations in glucocorticoids while caloric restriction, with its vastly reported benefits in the literature, leads to elevations in glucocorticoids, the “glucocorticoid paradox of caloric restriction” has been proposed (Patel and Finch, 2002). Indeed, knocking down the elevation of glucocorticoids during caloric restriction has been shown to lead to even higher neuroprotection. This suggests that benefits of caloric restriction may not be mediated by elevations of glucocorticoids.

Insulin and glucagon work in tandem to balance blood glucose levels within the body. Likewise, various studies have shown glucagon and insulin to share similar mechanisms of neuroprotection (Fanne et al., 2011). In a rodent model of ischemic brain injury, insulin and glucagon improved poststroke outcome in animal models by decreasing glutamate in the circulation and cerebrospinal fluid (Campos et al., 2011). In our model, 14 h of CR led to higher serum corticosterone levels and lower glucagon and insulin levels. We postulate that the increase in corticosterone may be a downstream effect of very short-term dietary-induced stress, which may mediate some form of preconditioning. Moreover, the decrease in glucagon and insulin levels may be a potential reflection of the onset of glycemic normalization in the CR rats, and may loosely suggest that neither are directly involved in the yielded effects of a 14 h period of CR. The intertwining and role of these metabolites in the grander mechanisms of overnight CR remains nebulous and warrants careful examination.

### SIRT-1 and BDNF

SIRT1 is a NAD+-dependent deacetylase which has a multitude of downstream effects, including inhibition of NF-kB, protection from oxidative stress, and a regulator of autophagy. SIRT1 has also been shown to possess neuroprotective properties in a variety of pathological conditions including neurodegenerative diseases and cerebral ischemia. Moreover, cerebral ischemia injury has been shown to be attenuated by short-term caloric restriction via upregulation of SIRT1 expression (Manzanero et al., 2011; Ran et al., 2015). Despite these overwhelming studies in lengthier CR experiments, our results revealed no significant difference in the upregulation of SIRT1 following a 14 h period of CR.

BDNF is a neurotrophic factor with a wide variety of immediate and long-term effects. It exerts immediate effects by altering synaptic transmission, while exerting longer term effects via synaptogenesis and neurogenesis (Zuccato and Cattaneo, 2009). Through these mechanisms, BDNF and its receptor tropomyosin-related kinase B (TrkB) provide neuroprotection against stress and cell death. Long-term CR is known to have a multitude of beneficial effects, including protection against age-related cognitive decline (Manzanero et al., 2011). Increased BDNF expression has been shown to mediate many of these effects (Kishi et al., 2015). Likewise to our findings with SIRT1, our results yielded no significant difference in BDNF expression following such a transient period of CR. We therefore suggest that the mechanisms involved in such an ultra-short overnight CR are independent of those afforded by SIRT-1 and BDNF, contrary to the supporting literatures that investigate lengthier periods of CR.

### Implications for Basic Science Research

In the clinical setting, overnight fasting is implemented preoperatively in attempt to prevent pulmonary aspiration of digestive content during endotracheal intubation and while under the effects of general anesthesia. This practice has been standardized, as even the most minor cases of patient aspiration could lead to aspiration pneumonitis, pneumonia, and respiratory failure. In animal models of experimental injury, however, preoperative overnight fasting is not always implemented. In rodent models, justification for this is in part due to a lack of emetic reflex in mice and rats, which diminishes aspiration-related complications (Horn et al., 2013). Yet, reports about laboratory practices the night prior to experimentation range from *ad lib* diet, overnight fasting, to being unspecified in the materials and methods of published studies. In rodent cardiac arrest models specifically, some studies report complete *ad lib* diet (Katz et al., 1995; Dohi et al., 2006; Farkas et al., 2010; Shoykhet et al., 2012), while others report fasting the night prior to experiments (Chen et al., 2013; Gong et al., 2015; Yang et al., 2015), and yet others do not specify this information (Kawai et al., 1992; Xie et al., 1995; Schmitz et al., 1997; Schurr et al., 2001). Transient dietary modifications have remarkable effects on various experimental parameters, particularly in models of behavior (Gainey et al., 2016; Eudave et al., 2018; Vega-Torres et al., 2020) and brain injury (Wu et al., 2003; Prins, 2008; Li et al., 2013). Our findings support that pre-experimental dietary variables have a major impact on outcome which may, if unintended in experimental design, contribute to irreproducibility of results. Accordingly, we suggest that information on pre-injury dietary variables should be required in “Methods” sections.

## Limitations

Although we investigated some candidate markers in select pathways previously shown to be important in CR and various models of cerebral ischemia, our study does not examine these metabolites after cardiac arrest nor the multitude of other potential pathways and metabolites that could be affected by ultra-short caloric restriction. In addition, only male rodents were utilized in our study. This is a major limitation given that there exists a multitude of sex differences between males and females in cardiovascular disease, including myocardial infarction, that warrant further investigation in the context of our study (Regitz-Zagrosek et al., 2016). This is also of relevance because while stroke is more common in males, it tends to have higher rates of morbidity and mortality in females (Appelros et al., 2009). Furthermore, our study only utilized one behavioral assessment (neurological deficit scale score). Additional behavioral tests that include cognitive, memory, and exploratory tasks (i.e., object recognition, swim-tests, and open-field test) could have been utilized to further evaluate precise deficits and recovery (Schaar et al., 2010). Another potentially important biological variable is time of day, particularly because metabolic parameters vary over the light-dark cycle. Generalizing our findings to humans is also uncertain because rodents are nocturnal whereas humans are diurnal. Rodents also have significantly higher metabolic rates than larger animals, and previous studies have reported that overnight CR of rodents has different consequences than overnight CR in humans (Jensen et al., 2013). Moreover, while we measured levels of hormones and metabolites after overnight CR but immediately before CA, the levels of these components post-resuscitation also warrant investigation as they may have an important role in neurological outcome. Further studies (i.e., transcriptomics/metabolomics) will be required, including assessment of factors post-resuscitation, to parse out which factors actually mediate neuroprotection.

## Conclusion

To our knowledge, this is the first study focusing on ultra-short CR (<24 h) in an ischemic brain injury model. We demonstrate that a mere 14 h period of overnight caloric restriction prior to cardiac arrest and resuscitation in a rodent model has significant effects on survivability and neurological recovery, as supported by behavioral assessments, quantitative EEG metrics, as well as histological and blood serum analyses. Remarkably, our histological assessments show up to complete neuroprotection in certain locales of the brain. These findings motivate future studies to pinpoint the specific mechanisms of such a potent ultra-transient caloric restriction and spotlights the critical necessity of standardizing pre-experimental dietary methodologies to ensure research homogeneity and reproducibility.

## Data Availability Statement

The raw data supporting the conclusions of this article will be made available by the authors, without undue reservation, to any qualified researcher.

## Ethics Statement

The animal study was reviewed and approved by the University of California Animal Care Committee.

## Author Contributions

MA, GT, OS, and YA planned the experiments, tested the data statistically, and wrote the manuscript. MA, GT, MD, IO, FA, SZ, AK, YS, and MW conducted the *in vitro* experiments, including molecular and histological procedures. AB, NM, MR, and YA conducted the *in vivo* experiments, including CA and surgical procedures. NC and BL conducted ECoG data analyses. All authors contributed to the article and approved the submitted version.

## Conflict of Interest

The authors declare that the research was conducted in the absence of any commercial or financial relationships that could be construed as a potential conflict of interest.

## Supplementary Material

Supplementary Table 1 Arterial blood was collected and analyzed in both control and CR groups 10 min prior to CA and 10 min after return of spontaneous circulation (post-CA), reported as mean ± SD. Two-way repeated measures ANOVA revealed no significant difference between groups or time in pre- or post-CA arterial blood gas measurements (e.g., pH, CO_2_, O_2_, electrolytes, etc.) or hemodynamic data. CPR duration is total time (not including 8 min of asphyxia) of compression until return of spontaneous circulation. Time-to-CA is defined as total time until systolic blood pressure drops to <30 mmHg and pulse pressure drops to <10 mmHg. MAP, mean arterial pressure.
