# Supplementary material for: Overnight Caloric Restriction Prior to Cardiac Arrest and Resuscitation Leads to Improved Survival and Neurological Outcome in a Rodent Model
Source: Front Neurosci. 2021 Jan 12;14:609670. doi: 10.3389/fnins.2020.609670 (PMC7835645; doi:10.3389/fnins.2020.609670)
Supplement: Supplementary Table 1 — Arterial blood was collected and analyzed in both control and CR groups 10 min prior to CA and 10 min after return of spontaneous circulation (post-CA), reported as mean ± SD. Two-way repeated measures ANOVA revealed no significant difference between groups or time in pre- or post-CA arterial blood gas measurements (e.g., pH, CO2, O2, electrolytes, etc.) or hemodynamic data. CPR duration is total time (not including 8 min of asphyxia) of compression until return of spontaneous circulation. Time-to-CA is defined as total time until systolic blood pressure drops to <30 mmHg and pulse pressure drops to <10 mmHg. MAP, mean arterial pressure. [file Data_Sheet_1.PDF]

Supplemental Figures

Arterial Blood Measurement	Control *Pre-CA*	CR *Pre-CA*	Control *Post-CA*	CR *Post-CA*	Significance
pH	7.42 ± 0.1	7.39 ± 0.1	7.40 ± 0.04	7.43 ± 0.1	None, P>0.05
CO_2 (mmHg)	38.3 ± 7.7	39.1 ± 7.5	38.9 ± 7.6	39.2 ± 6.1	None, P>0.05
O_2 (mmHg)	166.4 ± 34.5	165.42 ± 24.5	164.9 ± 32.5	169.5 ± 55.7	None, P>0.05
HCO_3^- (mmol/L)	24.9 ± 2.2	25.9 ± 2.2	25.9 ± 2.1	26.0 ± 2.2	None, P>0.05
Sodium (mmol/L)	139.7 ± 2.3	140.1 ± 3.0	139.1 ± 2.5	139.2 ± 1.2	None, P>0.05
Potassium(mmol/L)	3.7 ± 0.4	3.8 ± 0.5	3.6 ± 0.6	4.0 ± 0.3	None, P>0.05
Calcium(mmol/L)	1.37 ± 0.1	1.34 ± 0.1	1.37 ± 0.1	1.38 ± 0.1	None, P>0.05
Hemoglobin (g/dL)	11.8 ± 0.9	11.5 ± 0.8	11.9 ± 0.7	12.0 ± 1.2	None, P>0.05
**Hemodynamic Measurement**	**Control** ***Pre-CA***	**CR** ***Pre-CA***	**Control** ***Post-CA***	**CR** ***Post-CA***	**Significance**
Time-to-CA (min)	2.65 ± 0.7	2.61 ± 0.7	—	—	None, P>0.05
CPR Duration (sec)	—	—	46.3 ± 16.6	54.5 ± 26.8	None, P>0.05
MAP (mmHg)	89.31 ± 11.7	91.44 ± 10.1	81.6 ± 17.1	83.2 ± 19.9	None, P>0.05
Heart Rate (bpm)	417 ± 40.9	411 ± 51.2	406 ± 44.7	409 ± 39.6	None, P>0.05

Table S1. Arterial blood was collected and analyzed in both control and CR groups 10 minutes prior to CA and 10 minutes after return of spontaneous circulation (post-CA), reported as mean ± SD. Two-way repeated measures ANOVA revealed no significant difference between groups or time in pre- or post-CA CA arterial blood gas measurements (e.g. pH, CO2, O2, electrolytes, etc.) or hemodynamic data. CPR duration is total time (not including 8 minutes of asphyxia) of compression until return of spontaneous circulation. Time-to-CA is defined as total time until systolic blood pressure drops to < 30 mmHg and pulse pressure drops to < 10 mmHg. MAP, mean arterial pressure.
